# Long-term clinical outcomes and predictive factors in patients with chronic ocular graft-versus-host disease

**DOI:** 10.1038/s41598-022-17032-2

**Published:** 2022-07-29

**Authors:** Hyeon-Jeong Yoon, Ga-Young Song, Kyung Chul Yoon

**Affiliations:** 1 Department of Ophthalmology, Chonnam National University Medical School and Hospital, 42 Jebong-ro, Dong-gu, Gwangju, 61469 Republic of Korea; 2 Department of Hematology-Oncology, Chonnam National University Hwasun Hospital, Hwasun, Jeollanamdo Republic of Korea

**Keywords:** Eye diseases, Corneal diseases, Graft-versus-host disease

## Abstract

We investigate long-term clinical outcomes and predictive factors associated with poor vision outcomes in patients with ocular graft-versus-host disease (oGVHD). This retrospective cohort study involved 94 patients with chronic oGVHD, classified into severe (n = 25) and non-severe (n = 69) groups. Factors associated with oGVHD severity and poor vision outcomes were examined using multivariate logistic regression. In the severe oGVHD group, the disease activity pattern tended to be persistent, whereas flare-up episodes were more frequent and occurred over shorter intervals in this group. Myelodysplastic syndrome (MDS) and lung GVHD were more common and systemic calcineurin inhibitors were used more frequently in the severe group than in the non-severe group. Finally, 5-year survival rates were poorer in the severe group. Multivariate analysis revealed that MDS, lung GVHD involvement, and no history of systemic calcineurin inhibitor use were risk factors for severe oGVHD. Risk factors for poor vision outcomes were conjunctival scarring and persistent epithelial defects. In conclusion, MDS, lung GVHD, and no history of systemic calcineurin inhibitors are associated with severe oGVHD. Conjunctival scarring and persistent epithelial defects are risk factors for poor vision outcomes.

## Introduction

Chronic graft-versus-host disease (GVHD) is a potentially life-threatening complication of allogeneic hematopoietic stem cell transplantation (HSCT), a treatment for hematologic disorders as well as hematologic or lymphoid malignancies^1,2^. Chronic GVHD is an immune-mediated disorder that can target multiple organs including the skin, mucosa, liver, and lung^1–3^. Ocular involvement is observed in 60–90% of patients with GVHD and primarily presents with severe dry eye (DE)^2,4–6^. The pathophysiology of ocular GVHD is not fully understood, however, it is recognized as a complex interaction between donor immune cells and host histocompatibility antigens involving multiple inflammatory cascades and molecular mechanisms including pathogenic fibrosis and stress-induced aging^2,7^. These interactions lead to an inflammatory environment that causes impaired epithelial integrity of the ocular surface and the destruction and fibrosis of the conjunctiva and lacrimal gland^8,9^. Together, these inflammatory and biomechanical changes may lead to secondary complications including resulting in blindness and loss of vision-related quality of life^10–12^.

The diagnostic criteria for chronic oGVHD have been proposed by the National Institute of Health (NIH) consensus or International Chronic oGVHD Consensus Group (ICOGCG)^1,13–16^. While these criteria are useful for diagnosis, they preclude meaningful prognostication of vision outcomes, as they refer to DE parameters and subjective symptoms only. Many vision-threatening ocular manifestations (e.g. corneal neovascularization and melting perforation) associated with oGVHD have been reported in the literature; however, only few studies have focused on these severe ocular complications^9,17,18^. Several previous studies have examined risk factors associated with the occurrence of oGVHD, such as prior acute GVHD, peripheral blood stem cells, gender of donor, absence of anti-thymocyte globulin prophylaxis, number of systemic organ involvement, and non-Caucasian. However, there were no studies about risk factors associated with severe ocular complications in oGVHD patients^9,18^.

The management of vision-threatening complications associated with oGVHD is often challenging, due to severe inflammation, lack of vital components to maintain ocular surface homeostasis, and poor systemic condition^7,19–21^. Identification of risk factors associated with oGVHD severity and poor vision outcomes may support the clinical management of affected patients. Herein, we aimed to investigate long-term clinical outcomes in patients with chronic oGVHD and to identify risk factors for severe chronic oGVHD and poor vision outcomes.

## Results

This study included 94 patients with chronic oGVHD. The mean age was 40.93 ± 16.62 years; 55 (58.5%) patients were men. The mean interval duration between HSCT and disease onset and total follow-up duration were 521.9 ± 501.3 and 1641.6 ± 1320.9 days, respectively.

Table 1 showed the clinical ophthalmic features of patients with chronic oGVHD at the time point of the most severe ocular status during follow-up periods. Decreased tear volume, conjunctival hyperemia, meibomian gland dysfunction, and superficial punctate keratitis were present in over 80% of the patients. Most (n = 71, 75.5%) patients showed severe ophthalmic symptoms with an NIH oGVHD score of 3 points.

**Table 1:** Clinical characteristics of patients with chronic ocular graft-versus-host disease.

Ocular complications	n (%)
Decreased tear volume (< 5 mm/5 min)	81 (86.2)
Conjunctiva hyperemia	85 (90.4)
Conjunctival scaring	37 (39.4)
Meibomian gland dysfunction	83 (88.3)
Superficial punctate keratitis	94 (100)
Filamentary keratitis	71 (75.5)
Persistent epithelial defects	33 (35.1)
Corneal thinning	4 (4.3)
Corneal neovascularization	8 (8.5)
Infection history	14 (14.9)
NIH ocular score*	
Grade 0–1	0 (0)
Grade 2	23 (24.5)
Grade 3	71 (75.5)

*NIH* National Institute of Health.

Table 2 shows the demographic and clinical characteristics of the patients in the severe (n = 25) and non-severe (n = 69) chronic oGVHD groups. MDS (28.0% vs. 7.2%) and lung GVHD (56.0% vs. 33.3%) were more frequent in the severe group than in the non-severe group (*p* = 0.014 and *p* = 0.041, respectively). Both groups were comparable in age, sex, transplantation characteristics, and acute GVHD history (all *p* > 0.05).

**Table 2:** Differences in systemic characteristics between the severe and non-severe graft-versus-host disease (GVHD) groups.

	Severe oGVHD group (n = 25)	Non-severe oGVHD group (n = 69)	*p*-value
Age (years)	45.92 ± 15.59	39.12 ± 16.72	0.073
Sex (M:F)	17:8	38:31	0.188
**Causative disease, n**			0.069
Acute myeloid leukemia	6	29	0.149
Acute lymphoblastic leukemia	6	25	0.326
Myelodysplastic syndrome	7	5	0.014
Lymphoma	4	4	0.202
Others	2	5	0.999
**Transplantation, n**			
Stem cell sources (PBSCT : BMT)	22:3	64:5	0.360
Donor Sibling-unrelated-haploidentical	12–11-1	37–26-5	0.762
Sex match: sex mismatch	13:9	31:36	0.213
HLA matching	7.48 ± 1.03	7.43 ± 1.10	0.868
Pre-transplantation management (Chemotherapy : Chemo + TBI)	18:5	49:17	0.468
Anti-thymocyte globulin prophylaxis	8:14	28:39	0.424
Acute GVHD history, n	10:11	32:36	0.580
**Systemic GVHD, n**			
Cutaneous (yes/no)	16/9	44/25	0.592
Oral (yes/no)	19/6	48/21	0.369
Gut (yes/no)	5/20	11/58	0.427
Liver (yes/no)	9/16	31/38	0.297
Lung (yes/no)	14/11	23/46	0.041
Others (yes/no)	1/24	9/60	0.194
Number of involved organs	2.75 ± 0.99	2.37 ± 1.01	0.382
Systemic GVHD grade	2.57 ± 0.51	2.40 ± 0.63	0.266
Duration of graft-to-eye (days)	482.52 ± 327.59	536.26 ± 452.36	0.649
Follow-up duration (days)	1324.32 ± 1125.78	1756.5 ± 1309.77	0.162

*PBSCT* Peripheral blood stem cell transplantation, *BMT* Bone marrow transplantation, *HLA* Human leukocyte antigen, *TBI* Total body irradiation.

The pattern of disease activity is summarized in Table 3. In the severe oGVHD group, the disease course tended to be persistent (*p* = 0.014) and flare-up episodes occurred more frequently (n = 0.034) in a short interval (n = 0.007), compared to those observed in the non-severe group.

**Table 3:** Pattern of disease activity during follow-up periods in the severe and non-severe ocular graft-versus-host disease (GVHD) groups.

	Severe oGVHD group (n = 25)	Non-severe oGVHD group (n = 69)	*p*-value
Persistent	12	15	0.014
**Episodic**			
Number of episodes	2.64 ± 1.99	1.60 ± 0.85	0.034
Interval (months)	8.56 ± 10.68	17.25 ± 14.69	0.007

*oGVHD* Ocular graft-versus-host disease.

Table 4 presents the results of systemic immunosuppressant use comparisons between the severe and non-severe oGVHD groups. There was no difference in the number of systemic immunosuppressants used between the groups. However, systemic calcineurin inhibitors were more frequently used in the non-severe group than in the severe group.

**Table 4:** Differences in systemic immunosuppressant for GVHD before ocular flare-up between severe and non-severe groups.

	Severe oGVHD group (n = 25)	Non-severe oGVHD group (n = 69)	*p*-value
Number of systemic immunosuppressant	2.60 ± 1.19	2.57 ± 1.25	0.904
**Class of immunosuppressants**			
Corticosteroids	23	61	0.329
Antimetabolites (e.g., MTX, MMF, azathioprine)	13	38	0.443
Calcineurin inhibitor (e.g., cyclosporine, tacrolimus)	13	57	0.035
Alkylating agents (e.g., cyclophosphamide)	2	2	0.278
JAK2 inhibitor	3	6	0.431
TNF-α blocker	0	3	0.399
Tyrosine kinase inhibitor	3	5	0.346

*MTX* Methotrexate, *MMF* Mycophenolate mofetil, *JAK* Janus kinase, *TNF* Tumor necrosis factor, *oGVHD* Ocular graft-versus-host disease.

Predictive factors associated with severe oGVHD (score of ≥ 15 points) and poor final vision outcomes (best-corrected visual acuity [BCVA] of < 20/200) derived from logistic regression analysis are shown in Table 5. Severe oGVHD was significantly associated with the presence of MDS (odds ratio [OR], 5.50, p = 0.019) and lung GVHD (OR, 4.17, *p* = 0.041). No history of systemic calcineurin inhibitor use was significantly associated with severe oGVHD (OR, 3.95, *p* = 0.047). Poor vision outcomes were associated with the presence of conjunctival scarring (OR, 4.04, *p* = 0.045) and persistent epithelial defects (PED; OR, 14.76, *p* < 0.001).

**Table 5:** Factors associated with severe ocular graft-versus-host disease (GVHD) and poor vision outcomes.

Variables	Univariate analysis		Multivariate analysis	
	Odds ratio	*P*-value	Odds ratio	*P*-value
**Severe ocular GVHD**				
Sex (male/female)	1.091	0.272		
Myelodysplastic syndrome (yes/no)	7.271	0.007	5.500	0.019
Donor (sex match/sex mismatch)	1.677	0.299		
Anti-thymocyte globulin prophylaxis	1.750	0.653		
Acute GVHD history (yes/no)	1.023	0.964		
Lung GVHD (yes/no)	4.432	0.035	4.165	0.041
Systemic GVHD grade	1.248	0.264		
No history of systemic calcineurin inhibitor	4.591	0.032	3.952	0.047
**Poor vision outcomes**				
Myelodysplastic syndrome (yes/no)	8.784	0.009	0.907	0.341
Lung GVHD (yes/no)	4.015	0.045	0.465	0.495
Tear volume (< 5 mm/min/or not)	0.270	0.603		
Conjunctival scaring (yes/no)	4.015	0.045	4.036	0.045
Conjunctival hyperemia (yes/no)	1.496	0.221		
MGD (yes/no)	1.683	0.195		
Superficial punctate keratitis (> 4/ < 4)	8.090	0.004	1.699	0.199
Filamentary keratitis (yes/no)	4.600	0.032	1.705	0.197
Persistent epithelial defects (yes/no)	30.149	< 0.001	14.757	< 0.001
No history of systemic calcineurin inhibitor	0.007	0.936		

*CsA* Cyclosporin, *MGD* Meibomian gland dysfunction.

**Figure 1:**
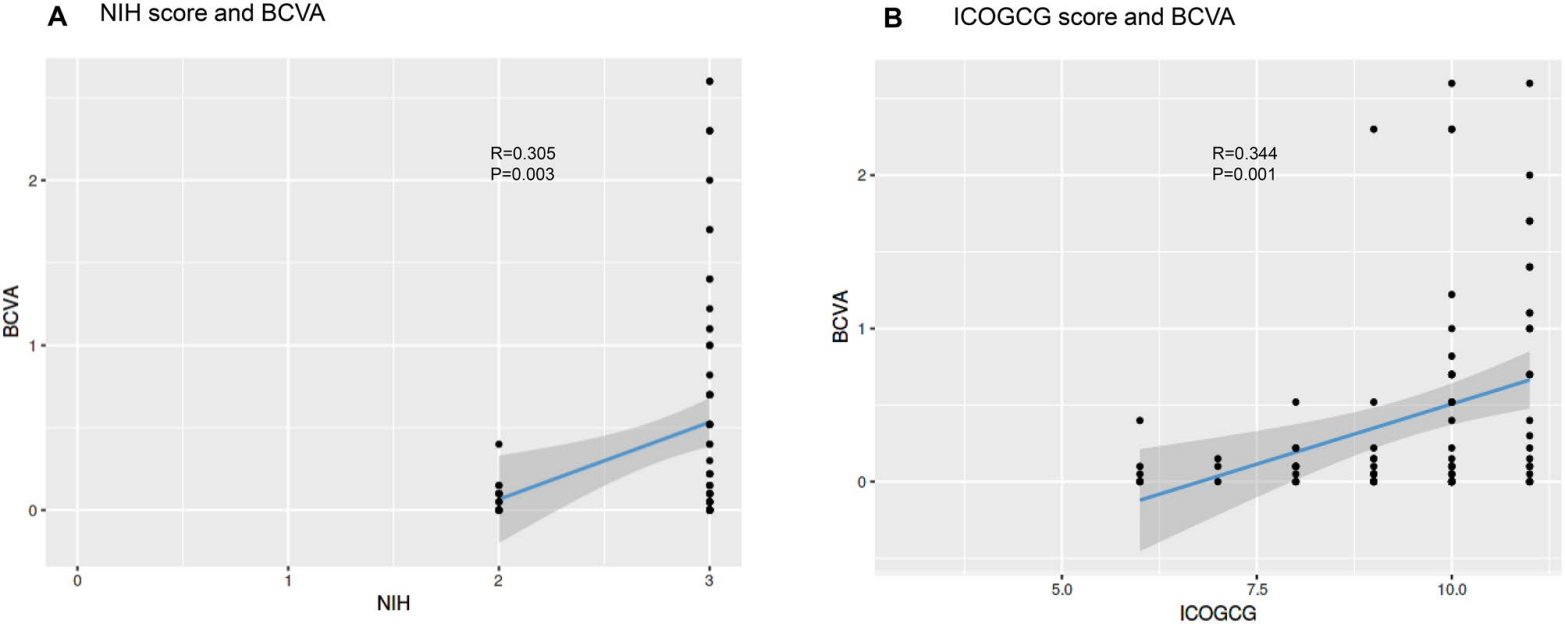
Correlation between final best-corrected visual acuity (BCVA), National Institute of Health (NIH) ocular scores, and International Chronic Ocular Graft-versus-Host-Disease (GVHD) Consensus Group (ICOGCG) score.

We examined the relationship between the scoring systems and final BCVA (Fig. 1). All scoring systems showed a significant correlation with BCVA (NIH: R = 0.305, *p* = 0.003; ICOGCG: R = 0.344, *p* < 0.001). In addition, we examined the relationship between the ICOGCG and systemic GVHD grade. There was no significant correlation (ICOGCG-systemic GVHD score: R = 0.02, *p* = 0.874).

**Figure 2:**
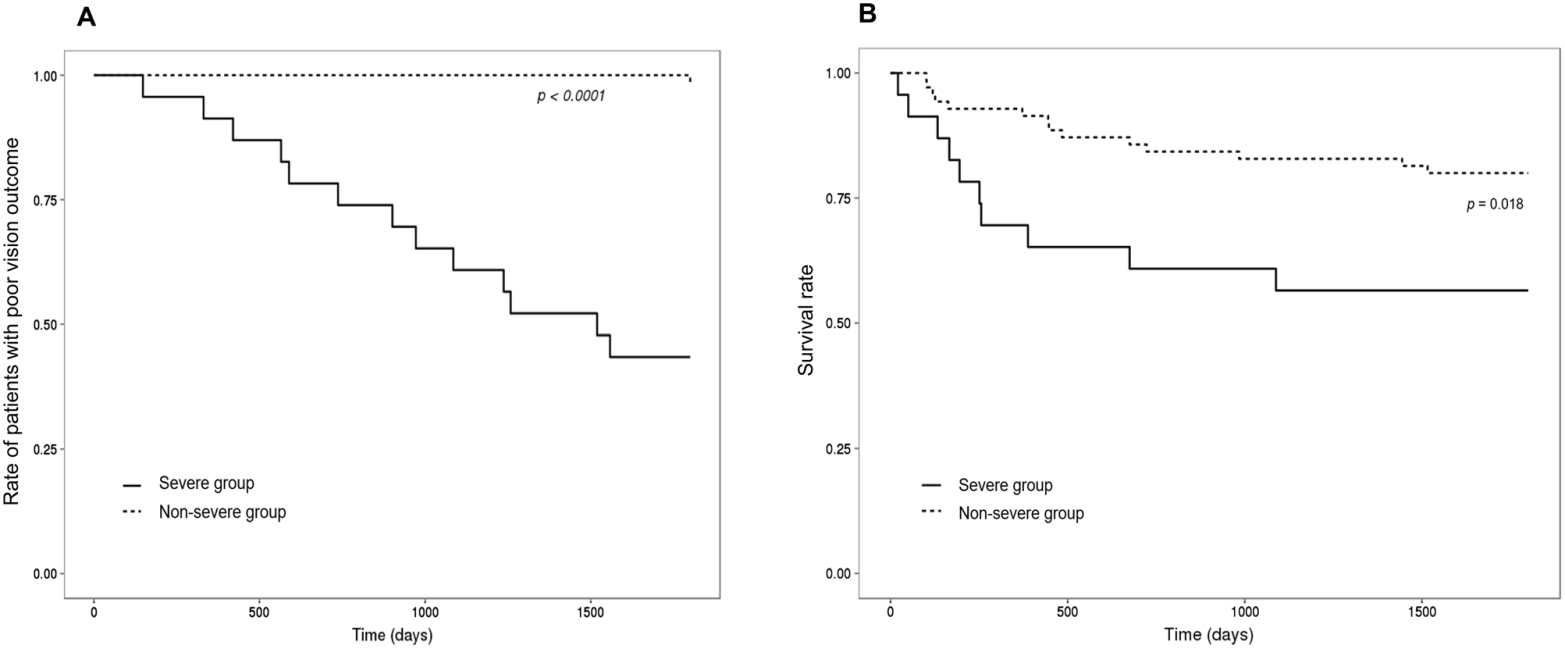
Five-year Kaplan–Meier survival curves of 94 patients with chronic ocular graft-versus-host disease (GVHD) for (**A**) poor vision outcomes and (**B**) overall survival according to the severe and non-severe groups.

The Kaplan–Meier method revealed that 5-year rates of poor vision outcomes were higher in the severe oGVHD group than in the non-severe oGVHD group (*p* < 0.001). In addition, the 5-year survival rates were significantly lower in the severe oGVHD group than in the non-severe oGVHD group (*p* = 0.018; Fig. 2A and B).

## Discussion

Many patients with oGVHD suffer from severe ocular complications resulting in impairment of vision and quality of life^10,11^. The management of vision-threatening complications associated with oGVHD is often challenging^7,19–21^. Although previous studies on chronic oGVHD have reported clinical manifestations and associated risk factors^2,4,5,22–24^; some of these studies examined only one of the severe ocular manifestations, such as PED and corneal perforation^9,18,25^. To the best of our knowledge, this study is the first to report the risk factors for long-term clinical outcomes and poor final visual prognosis in oGVHD patients.

In the present study, patients with an NIH score of 3 points represented 75% of the sample, and the present rates of PED or infection were higher than those reported previously^9,18^. The present patients had been referred from the hematology department; most of the patients complained of ocular discomforts and voluntarily underwent ophthalmic care. Moreover, we retrospectively enrolled only the patients who met the ICOGCG criteria for definite ocular GVHD in this study. Therefore, our enrolled patients showed ocular findings more severe than those reported in the previous studies.

In addition, the long-term pattern of disease activity observed in this study was classified as persistent or episodic pattern for patients with chronic oGVHD. This study is first to propose this classification of disease patterns. During the mean follow-up period of 1324.32 ± 1125.78 days, we observed some patients with disease intensity fluctuations and others with sustained disease. In the severe oGVHD group, disease patterns tended to be persistent and flare-up episodes were common and frequent.

In this study, MDS, lung GVHD, and no history of systemic calcineurin inhibitors were associated with severe oGVHD. Conjunctival scarring and PED were risk factors for poor vision outcomes. The following characteristics are reportedly associated with the risk of chronic oGVHD: non-Caucasian ethnicity, prior history of acute GVHD, peripheral blood stem cell transplantation from a female donor to a male recipient or an Epstein-Barr virus-seropositive donor, no anti-thymocyte globulin prophylaxis, and other systemic GVHD^22,26,27^.

Several studies have reported an association between the causative disease and systemic GVHD and chronic oGVHD^24,27,28^. Hematological disorders including acute myeloid leukemia, acute lymphoblastic leukemia, and MDS were risk factors for DE in patients undergoing HSCT^28^. In this study, MDS was associated with severe oGVHD. MDS is a heterogeneous disease group of clonal stem cell disorders with a poor prognosis; currently, HSCT is the only option for MDS^29–31^. Owing to the lack of an anti-MDS treatment strategy with minimal toxicity, hematologists likely aimed at enhancing the graft-versus-leukemia effect, rather than considering GVHD^30^. In addition, sub-analysis showed that patients with MDS tended to have a great number of GVHD-involved organs than patients without MDS (2.75 ± 1.06 vs. 2.29 ± 1.04, *P* = 0.15).

In addition, the relationship between systemic and ocular GVHD was investigated in this study. Acute and chronic cutaneous involvements are known predisposing factors for the occurrence of chronic oGVHD^26,28,32^. Na et al.^28^ reported that GVHD in the oral mucosa and lungs was associated with the chronic stage of oGVHD. In this study, there were no direct correlation between systemic GVHD grade and ocular GVHD severity. However, lung GVHD was a risk factor for severe oGVHD. Lung GVHD is associated with a pathophysiological mechanism of epithelial-mesenchymal transition comparable to that observed in the eye^28,33^. In particular, the conjunctiva could mirror the systemic mucosal membranes^28,34^. In this study, conjunctival scarring was one of the risk factors for poor vision outcomes, suggesting that conjunctival findings may have key implications in the prognosis of GVHD.

Moreover, no history of systemic calcineurin inhibitor use was a risk factor for severe oGVHD. Calcineurin inhibitors, including cyclosporine and tacrolimus, interfere with T lymphocytes which are involved in chronic GVHD^35,36^. Although limited studies have been conducted on the use of systemic immunosuppressants for ocular surface disease, the effectiveness of systemic cyclosporine, one of the calcineurin inhibitors, is relatively well-established^37^. Several case studies have reported successful treatment using oral cyclosporine for ocular surface inflammatory disorders including limbal stem cell deficiency (LSCD), necrotizing scleritis, severe keratoconjunctivitis, and corneal graft rejection^37–41^. Several studies have reported that the initiation of topical cyclosporine in the pre-transplantation phase could reduce the inflammatory response in the lacrimal glands that may be responsible for the development of keratitis sicca^35,42^. They proposed that topical cyclosporine treatment in the early phase could achieve greater tissue levels of CsA and possibly forestall inflammatory damage to the lacrimal acini in GVHD^35^. Considering the ocular distribution of systemic calcineurine inhibitors^43,44^, our findings suggest that these immunosuppressants in the early phase of HSCT may be delivered to vascular-rich ocular tissues including the conjunctiva, and may help prevent ocular complications.

In this study, the 5-year survival rate was significantly lower in patients with severe oGVHD than in those with non-severe oGVHD. Jacobs et al.^27^ showed that median survival was poorer for patients with oGVHD than for those without oGVHD. Jabs et al.^34^ described a conjunctival staging system that reflects the severity of systemic GVHD associated with poorer survival. They reported that 5 of 44 patients with chronic GVHD had pseudomembranous conjunctivitis and 4 of 5 patients died within a mean of 352 days. Allan et al.^45^ reported that conjunctival scarring could be associated with the survival rates of patients with GVHD, although these authors noted that extensive systemic disease involved in essential organs could affect outcomes. In our study, a large proportion of patients with severe oGVHD also had lung GVHD. Nevertheless, patients with severe ocular findings should be comprehensively assessed for their systemic condition.

This study has several limitations. This study had a retrospective design with a small sample size; data on the systemic status might be insufficient to calculate the correlation with ocular conditions, patients with systemic GVHD but no ocular GVHD could not be included in the analysis due to lack of follow-up. Additionally, pre-HSCT ocular status and treatment regimens, including topical eye drops and systemic immunosuppressants, were not controlled. As systemic medication was prescribed by hematologists, uncontrolled factors may have affected the presented estimates. Multicenter prospective cohort studies with large samples are required to validate the present findings.

These limitations notwithstanding, this study has several strengths. This study was the first to analyze the long-term clinical outcomes affecting the visual prognosis and survival rates in oGVHD patients. This study has also shown that severe oGVHD was associated with MDS, lung GVHD, and no history of systemic calcineurin inhibitor use. Conjunctival scarring and PED were risk factors for poor vision outcomes. Severe oGVHD may be associated with poor post-transplant survival. In conclusion, our results suggest that frequent and careful follow-up of patients with these risk factors is recommended for proper management of chronic oGVHD in the real-world setting.

## Materials and methods

### Ethics consideration

Ethical approval was obtained from the Chonnam National University Hospital Institutional Review Board (CNUHIRB-2021-328; Gwangju, South Korea), and the study protocol adhered to the guidelines of the Declaration of Helsinki. In this retrospective study, data were extracted from the patients’ medical charts and recorded using electronic case report forms. We explained the study information to each patient and obtained informed consent.

### Study patients

Patients with chronic oGVHD, who underwent evaluation between January 2013 and October 2020, were included in this study. A total of 300 patients referred for ophthalmological assessment from the hematology department were identified. Patients who did not undergo HSCT (n = 105) and those who visited the clinic within 100 days of HSCT (n = 20) were excluded. Patients without ocular GVHD (n = 65) and those with incomplete records (n = 16) were also excluded (Supplementary Fig. 1). A total of 94 patients included in this study fulfilled the ICOGCG diagnostic criteria for chronic oGVHD and were followed up for at least 1 year. The medical records of the enrolled patients were reviewed by a hemato-oncologist (G.Y.S.).

### Medical records

Age, sex, causative disease, acute GVHD history, interval between HSCT, disease onset, and follow-up duration were investigated. Data on allogeneic HSCT, including stem cell sources for HSCT (bone marrow or peripheral blood stem cell), relation with donor (sibling/unrelated/haploidentical and sex-matching), number of human leukocyte antigen matches, and pre-transplantation management (chemotherapy, total body irradiation and anti-thymocyte globulin prophylaxis) were extracted. Disease onset was based on the date of diagnosis of definite ocular GVHD. Other organs affected by chronic systemic GVHD were classified as cutaneous tissue, oral mucosa, gut, liver, lung, and other organs. Data on systemic agents for GVHD used in the early phase, such as corticosteroids, antimetabolites (mycophenolic acid, methotrexate, and azathioprine), calcineurin inhibitors (cyclosporine, tacrolimus), alkylating agents (cyclophosphamide), JAK2 inhibitors, tumor necrosis factor (TNF)-α blockers, and tyrosine kinase inhibitors were collected. For patients who died within 5 years of follow-up, the interval between chronic oGVHD onset and death was included in survival analysis.

### Diagnostic criteria of chronic oGVHD

According to the ICOGCG criteria, severity scores were assigned to the ocular surface disease index (OSDI; score 0, 0–13; score 1, 13–22; score 2, 23–32; score 3, ≥ 33), corneal fluorescein staining (score 0, no staining; score 1, minimal staining; score 2, mild/moderate staining; score 3, severe staining), conjunctival injection (score 0, none; score 1, mild/moderate; score 2, severe), and Schirmer’s score (score 0, > 15; score 1, 11–15; score 2, 6–10; score 3, ≤ 5)^14^. All scores were based on a representative image provided by the ICOGCG^14^. Definite oGVHD was diagnosed based on the scores of ≥ 6 points with systemic GVHD or ≥ 8 points without systemic GVHD^14^. Systemic GVHD and its grade were defined according to the 2014 NIH chronic GVHD consensus criteria^1^.

Severity was scored, according to the NIH scoring criteria for chronic oGVHD, as follows: 0 points, no symptoms; 1-point, mild DE symptoms not affecting any activities of daily living (ADL; requiring eyedrops or asymptomatic signs of keratoconjunctivitis sicca); 2 points, moderate DE symptoms partially affecting ADL (requiring drops > 3 times/day or punctal plugs) without vision impairment; and 3 points, severe DE symptoms significantly affecting ADL (special eyewear to relieve pain required) or resulting in an inability to work due to ocular symptoms or loss of vision caused by keratoconjunctivitis sicca^1^.

### Examination of ocular manifestations

Visual acuity, slit-lamp exam, and the schirmer test were performed at all visits. Ocular examination findings included the presence or absence of decreased tear volume, conjunctival hyperemia and scarring, meibomian gland dysfunction, superficial punctate keratitis, filamentary keratitis, PED, corneal thinning, and LSCD. Infection history included bacterial, fungal, or viral infections, and the NIH ocular score was investigated. Decreased tear volume was defined as < 5 mm/5 min using the Schirmer test without anesthesia. LSCD was defined as limbal flattening, corneal neovascularization, and/or a whorled vortex pattern in the epithelium. PED was considered a corneal epithelial defect persisting for at least 2 weeks, according to previous recommendations.

When definite ocular GVHD was present, follow-up was performed once a month. In cases requiring intensive care such as infectious keratitis, hospitalization was performed within 2 weeks. In remission state without definite ocular GVHD, follow-up was performed every 3 months (Supplementary Fig. 2).

### Pattern of disease activity

Disease activity was assessed by the presence or absence of definite ocular GVHD. An episodic pattern was defined as fluctuations in the intensity of disease activity (repeated remitting and relapsing) during the follow-up period. A persistent pattern was defined as continuous ocular manifestations, without remission, following disease onset. If the patients showed an episodic pattern, the number of episodes and duration of intervals were investigated during the follow-up period.

### Main outcome measurement

BCVA and the ocular manifestations were used to assess chronic ocular complications, which were the main outcome of interest. BCVA was obtained based on the time of no change in visual acuity for > 3 months at the final follow-up. Poor vision outcomes were defined as less than 20/200. Patients were classified into severe and non-severe groups according to the ocular manifestations of the most severe status during follow-up periods. The patients were classified as severe group, if two or more of the following ocular manifestations were present; persistent epithelial defects (attack > 3 times or persist 1 month), corneal thinning, corneal neovascularization, and infection history (viral, bacterial, or fungal). Twenty-five patients belonged to the severe group, and 69 patients belonged to the non-severe group.

### Statistical analysis

Statistical analyses were performed using the Statistical Package for the Social Sciences, version 22.0, for Windows (SPSS Inc., Chicago, IL, USA) and R version 3.4.3 (Rstudio, Inc, Boston, USA). The normality of distribution assumption was assessed using the Shapiro–Wilk test, and all the variables followed a normal distribution. The chi-square test was used to analyze the categorical data. All continuous variables were analyzed using the independent t-test. Univariate and multivariate logistic regression analyses were performed to investigate risk factors for severe chronic oGVHD and poor final vision outcomes. The Pearson correlation coefficient and Kaplan–Meier method were used to evaluate poor vision outcomes and overall survival and were performed using R software. Statistical significance was set at *P*-values of < 0.05.

## Supplementary Information

Supplementary Information 1.

Supplementary Information 2.

Supplementary Information 3.

## Data Availability

The datasets generated during the current study are available from the corresponding author on reasonable request.
