# Supplementary figures and images for: Long-term clinical outcomes and predictive factors in patients with chronic ocular graft-versus-host disease
Source: Sci Rep. 2022 Jul 29;12:12985. doi: 10.1038/s41598-022-17032-2 (PMC9338251; doi:10.1038/s41598-022-17032-2)

## Supplementary fig 1. Flow chart of patient eligibility

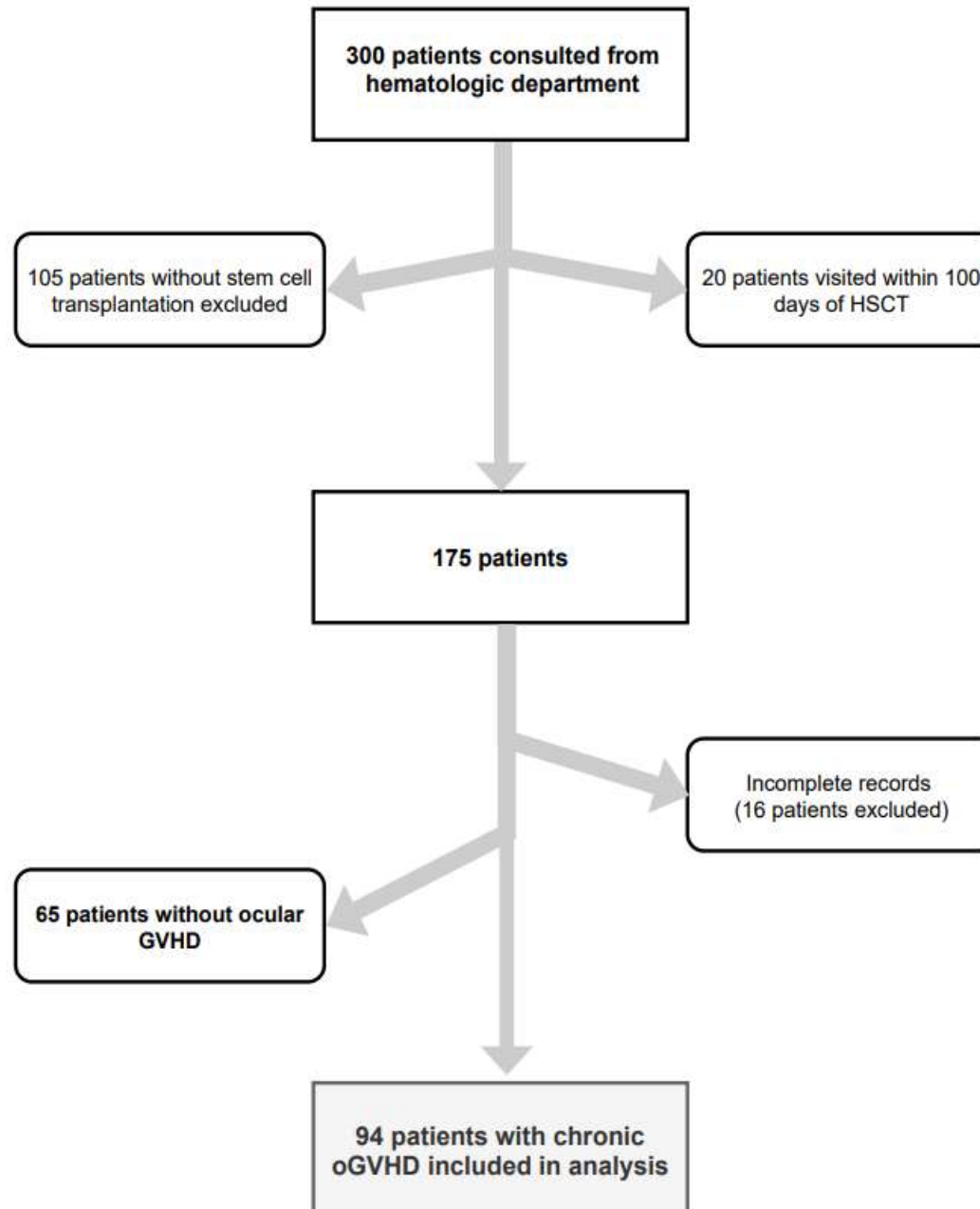

Supplement: Supplementary file 1 — Supplementary Information 1. [file 41598_2022_17032_MOESM1_ESM.pdf]
