# Supplementary material for: Long-term clinical outcomes and predictive factors in patients with chronic ocular graft-versus-host disease
Source: Sci Rep. 2022 Jul 29;12:12985. doi: 10.1038/s41598-022-17032-2 (PMC9338251; doi:10.1038/s41598-022-17032-2)

Supplementary fig 2. Time points of the follow-up examinations according to the disease patterns

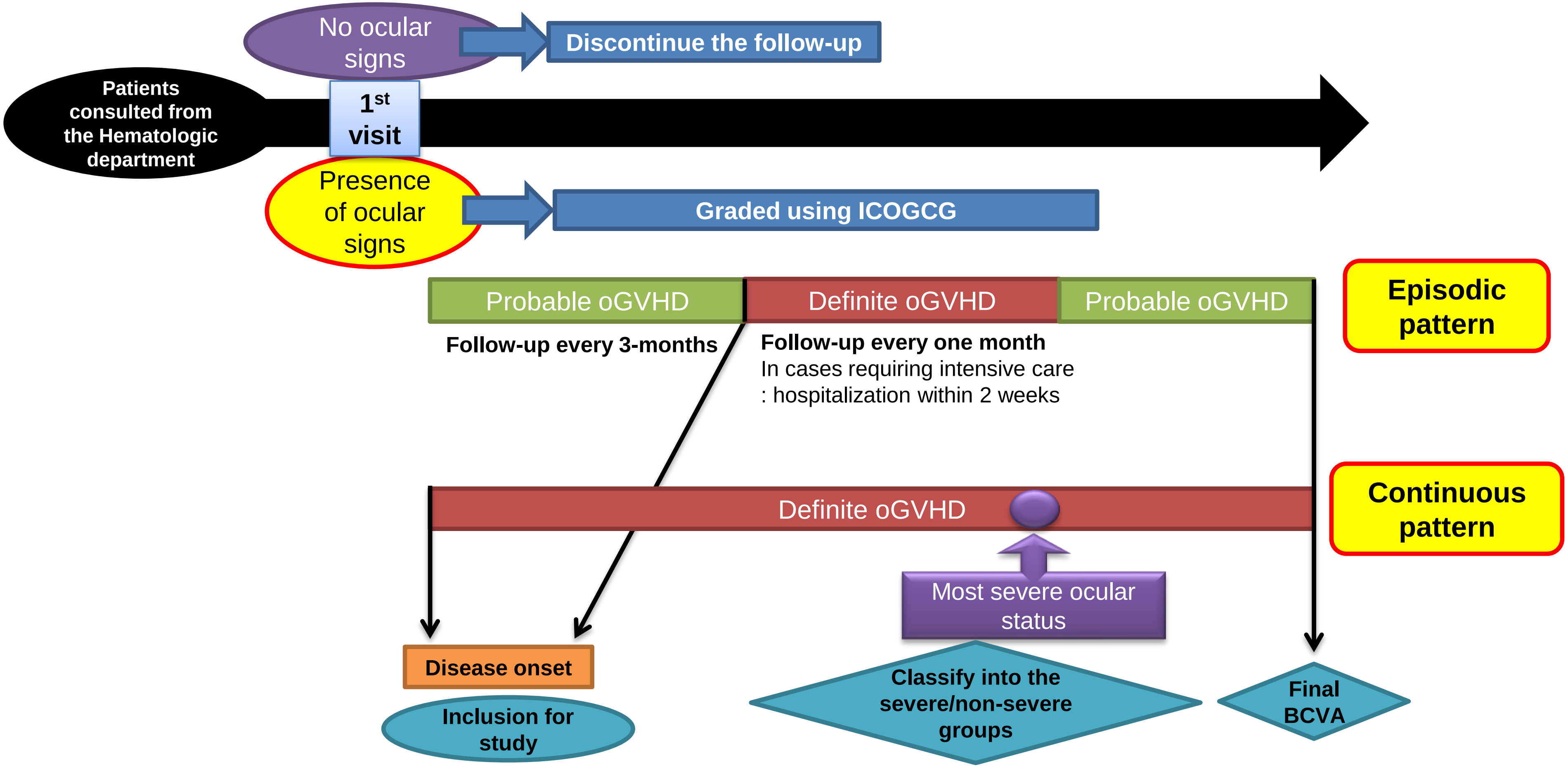

Supplement: Supplementary file 2 — Supplementary Information 2. [file 41598_2022_17032_MOESM2_ESM.pdf]
