# Supplementary material for: Long-term clinical outcomes and predictive factors in patients with chronic ocular graft-versus-host disease
Source: Sci Rep. 2022 Jul 29;12:12985. doi: 10.1038/s41598-022-17032-2 (PMC9338251; doi:10.1038/s41598-022-17032-2)

Supplementary fig 3. Anterior segment photography of a patient belonging to the severe ocular graft-versus-host disease group; (A) corneal superficial punctate keratitis and conjunctival hyperemia and scarring (6 months after disease onset); (B) infectious keratitis (1 year after disease onset); (C) near total corneal neovascularization (15 months after disease onset); (D) persistent epithelial defects (17 months after disease onset)

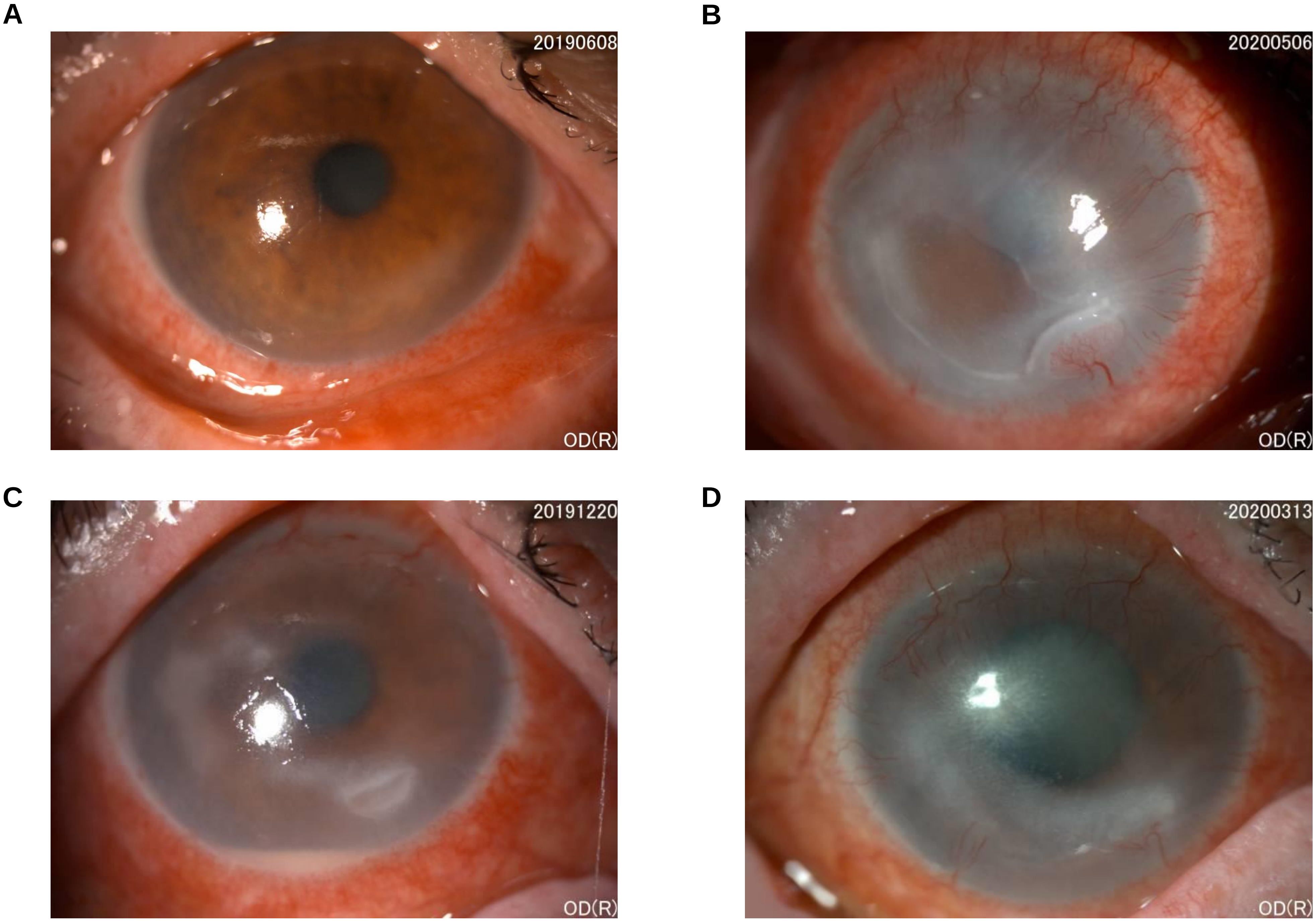

Supplement: Supplementary file 3 — Supplementary Information 3. [file 41598_2022_17032_MOESM3_ESM.pdf]
